# Modulation of Muscle Fiber Compositions in Response to Hypoxia via Pyruvate Dehydrogenase Kinase-1

**DOI:** 10.3389/fphys.2016.00604

**Published:** 2016-12-15

**Authors:** Daniel D. Nguyen, Gyuyoup Kim, Eung-Kwon Pae

**Affiliations:** ^1^Private PracticeSan Jose, CA, USA; ^2^Department of Obstetrics, Gynecology, and Reproductive Sciences, School of Medicine, University of MarylandBaltimore, MD, USA; ^3^Department of Orthodontics and Pediatric Dentistry, School of Dentistry, University of MarylandBaltimore, MD, USA

**Keywords:** fiber types, geniohyoid muscle, Hif-1, hypoxia, Pdk-1

## Abstract

Muscle fiber-type changes in hypoxic conditions in accordance with pyruvate dehydrogenase kinase (Pdk)-1 and hypoxia inducible factor (Hif)-1α were investigated in rats. Hif-1α and its down-stream molecule Pdk-1 are well known for readily response to hypoxia. We questioned their roles in relation to changes in myosin heavy chain (MyHC) composition in skeletal muscles. We hypothesize that the level of Pdk-1 with respect to the level of Hif-1α determines MyHC composition of the muscle in rats in hypoxia. Young male rats were housed in a chamber maintained at 11.5% (for sustained hypoxia) or fluctuating between 11.5 and 20.8% (for intermittent hypoxia or IH) oxygen levels. Then, muscle tissues from the geniohyoid (GH), soleus, and anterior tibialis (TA) were obtained at the end of hypoxic conditionings. After both hypoxic conditionings, protein levels of Pdk-1 and Hif-1 increased in GH muscles. GH muscles in acute sustained hypoxia favor an anaerobic glycolytic pathway, resulting in an increase in glycolytic MyHC IIb protein-rich fibers while maintain original fatigue-resistant MyHC IIa protein in the fibers; thus, the numbers of IIa- and IIb MyHC co-expressing fibers increased. Exogenous Pdk-1 over-expression using plasmid vectors elevated not only the glycolytic MyHC IIb, but also IIx as well as IIa expressions in C2C12 myotubes in ambient air significantly. The increase of dual expression of IIa- and IIb MyHC proteins in fibers harvested from the geniohyoid muscle has a potential to improve endurance as shown in our fatigability tests. By increasing the Pdk-1/Hif-1 ratio, a mixed-type muscle could alter endurance within the innate characteristics of the muscle toward more fatigue resistant. We conclude that an increased Pdk-1 level in skeletal muscle helps maintain MyHC compositions to be a fatigue resistant mixed-type muscle.

## Introduction

Increased fatigability of the tongue protruding muscles after intermittent hypoxia (IH) is associated with the pathophysiology of obstructive sleep apnea (OSA), one of the most prevalent global health problems (McSharry et al., 2012; Kim et al., 2014). Previously demonstrated is that a short-term IH challenge to growing rats results in changes of myosin heavy chain (MyHC) composition from IIa abundant to IIb dominant in the geniohyoid (GH) muscle, and which is accompanied by an increased fatigability (McGuire et al., 2002; Pae et al., 2005). This change in tongue muscles may explain a part of the pathophysiology of OSA. We think this change is initiated with an increased level of ubiquitous oxygen sensing molecule, hypoxia inducible factor (Hif)-1α in the muscle. Skeletal muscle is a dynamic and adaptive tissue to changes in oxygen level in tissue during contraction as well as in various environments. Contractile proteins in muscle constantly change their fiber composition in response to the level of oxygen, yet fiber types of a skeletal muscle are associated with Hif-1α expression in the muscle (Pisani and Dechesne, 2005). Thus, muscles appear to find a homeostatic balance between their functions and oxygen levels in the environment quickly and constantly. However, a molecule linking metabolic cue and mechanical cue in the muscle is still uncertain. We assume that pyruvate dehydrogenase kinase (PDK) may play such a role linking metabolic cue to compositional phenotypic changes in the muscle leading to changes of functional properties of the muscle.

PDK plays a gatekeeper for the TCA cycle controlling quantity of pyruvate feeding into cells via controlling the activity of pyruvate dehydrogenase (PDH) which converts pyruvate to acetyl-CoA. Among the four known isoforms, PDK-1 is a potent suppressor of PDH, yet is less influenced by blood glucose levels than PDK-4 (Peters et al., 2001), the most abundant isoform in skeletal muscle tissue. Therefore, quantifying PDK-1 in a muscle would be a reliable way to estimate adaptability of the muscle to hypoxic conditions independently from blood glucose levels. PDK-1, as a direct target gene of Hif-1, actively regulates the function of mitochondria in hypoxic condition by shunting pyruvate toward lactate, thus permitting continued glycolysis (Kim et al., 2006; Papandreou et al., 2006). As PDK-1 increases in skeletal muscles, production of harmful reactive oxygen species (ROS) decreases via bypassing mitochondrial biogenesis (Semenza, 2007). This mechanism may be a more economical control of energy consumption for the skeletal muscle in hypoxia and at the same time, in this way, muscles could reduce cellular oxygen requirement. Thus, in hypoxia, (1) PDK-1 encourages glycolytic metabolism, and (2) PDK-1 curtails ROS production in skeletal muscle. Assuming skeletal muscles maintain a homeostasis of PDK-1 concentration for the best efficiency of a muscle, when hypoxia perturbs the homeostasis, muscle fiber composition also changes to best accommodate the environment. To understand this survival tactic between metabolic and cytochemical adaptation in skeletal muscles, we assume that PDK-1 would play a significant role in fiber transformation. We suggest that a relative amount of PDK-1 with respect to Hif-1 plays a significant role in the phenotypic composition of fiber types (for instance, MyHC IIb dominant to IIa) in a mixed-type muscle.

Adaptive responses of skeletal muscle to hypoxia involve glucose metabolism initiated by elusive Hif-1α which regulates other signaling molecules (Mason and Johnson, 2007) for better energy efficiency and tissue survival. Under normal ambient air, Hif-1α level is higher in predominantly glycolytic fibers compared to predominantly oxidative fibers (Pisani and Dechesne, 2005; Lunde et al., 2011). To date, the mechanisms underlying fiber type changes in skeletal muscle under hypoxic condition in association with the level of PDK-1 and Hif-1α protein concentrations is not fully understood (Semenza, 2007; Wüst et al., 2009; Le Moine et al., 2011). We assume that Pdk-1 (from here, we will use Pdk-1 instead of PDK-1 for we are using rats) expression levels with respect to Hif-1α may determine the principal glucose metabolic pathway between glycolytic and oxidative in rodent skeletal muscles. As the first step to investigate this, we examined whether a quantitative relationship between Pdk-1, Hif-1α and myosin heavy chain (MyHC) composition does exist in rat muscles. If so, manipulating Pdk-1 levels would be technically easier than regulating tissue levels of Hif-1α that constantly changing depending on oxygen levels in the tissue.

## Materials and methods

### Ethical approval

The experimental protocols and animal care were approved by the Institutional Animal Review Committee at UCLA (ARC # 2003-125-12) and were in accordance with the National Institute of Health guide for the care and use of laboratory animals and the *in vivo* studies were performed at UCLA.

### Preparation of animals

In total, seventeen male *Sprague Dawley* rats weighing approximately 300–350 g (7 week old) were used for all experiments including single fiber analysis and Western blot assays. Of them, 4 animals were randomly selected for control and 7 animals were used for sustained hypoxia (3 for 15 h and 4 for 30 h). We, later, added 6 animals for intermittent hypoxia challenge (3 for 15 h and 3 for 30 h) to obtain muscle tissues for comparison purpose.

### Experimental conditions for animals

Animals were housed in a commercially-designed chamber (30 inches × 20 inches × 20 inches; Proox model 110, BioSpherix Instruments, Redfield, NY), with a modified control system and temperature range from 22 to 24°C. The chamber held two rat cages, and operated under a 12 h light/dark cycle (light phase from 6:00 a.m. to 6:00 p.m.) with food and water continuously available. O_2_ concentration was controlled by a cycle timer (model 4608, Artisan, Randolph, NJ), which regulated N_2_ delivery from tanks. Moment-to-moment levels of O_2_ concentration were quantified and adjusted by an O_2_ sensor system (Proox 110, BioSpherix, Redfield, NY). Carbon dioxide concentration, measured by an analyzer (Capstar-100, CWE Inc., Admore, PA), was maintained between 0 and 0.1%.

Animals were exposed to hypoxic conditions during the light phase for a maximum 7.5 h per day. The sustained hypoxia profile maintained between 13.5 and 9.5%, but stabilized most time at 11.5% oxygen (balanced by N_2_ gas), 7.5 total hours per day. Animals requiring more than 7.5 h were subject to hypoxic conditions on subsequent days during the light phase; e.g., an animal in the 15 h group would be exposed to hypoxia during the first day, and to an additional 7.5 h exposure the following day. During the dark phase, animals were kept outside of the chamber and breathed room air. Intermittent hypoxia alternating 20.8 and 10.3% oxygen every 4 min as previously used (Pae et al., 2005) was performed for reference purpose. The muscles aimed to analyze were geniohyoid, soleus, and tibialis anterior (TA) muscles. Fibers of the geniohyoid orient parallel to the long axis of the muscle, contractions displace the hyoid apparatus anteriorly, resulting in an enlarged pharyngeal lumen (Wiegand et al., 1990). In this way, geniohyoid muscles participate in breathing function.

### Tissue collection

At the end of the designated duration of hypoxic conditions, both control and experimental animals were sacrificed by overdose of pentobarbital (100 mg/kg, I.P.). The desired muscles then were removed and snap-frozen in isopentane and cooled by liquid nitrogen at −70°C. Muscle samples were preserved for single fiber gel electrophoresis and Western blot assays.

### Measurements of Hif-1α and Pdk-1 proteins in rat muscles

Harvested muscle tissues of approximately 150 mg obtained from 2 animals per group were equally partitioned and ground in a 1.5 ml Eppendorf tube. Ten volumes of RIPA extraction buffer (25 mM Tris•HCl pH 7.6, 150 mM NaCl, 1% NP-40, 1% sodium deoxycholate, 0.1% SDS) was added to the tissue. Following approximately 30 strokes of homogenization, samples were centrifuged for 10 min at 13,000 rpm at 4°C. All protein concentrations in the collected supernatant were determined with the detergent-compatible Bio-Rad DC protein assay using bovine serum albumin (BSA) as a standard.

For Western blotting, 50 μg of total proteins was heated at 95°C for 5 min and was separated by 4–12% gradient SDS-PAGE gel for Hif-1α and Pdk-1 proteins. The proteins were transferred 0.45 μm polyvinylindene difluoride membranes, which were stained with Ponceau red to determine whether the transfers were uniform. Then, the membranes were blocked with 5% weight to volume (w/v) non-fat dry milk in 1 × PBS with 0.1% Tween-20 for 1 h at room temperature. The membranes were incubated for 2 h at room temperature with anti-Hif-1α polyclonal antibody (1:500; Cell Signaling, Cat. #3716) and anti-Pdk-1 polyclonal antibody (1:500, Assay Designs, Cat. # KAP-PK112). Horseradish peroxide-conjugated goat-anti-rabbit antibody (1: 2000; Santa Cruz Biotechnology, Santa Cruz, CA) was used as for secondary antibody. For loading-control, β-Actin (1:500, Santa Cruz Biotechnology, Santa Cruz, CA) was used. Bands were visualized by incubating blots in SuperSignal West Femto solution (Pierce, Cat. # 34094) for 15 min in room temperature and the images were obtained in Bio-Rad ChemiDoc viewer (Bio-Rad, Hercules, CA). Relative density to β-Actin was measured using a densitometer. Density of each gel image was standardized to β-Actin. Muscle tissue harvested from 2 animals per subgroup was pooled for Western blot assays. Two gels per subgroup, thus density measurements from 6 gels were averaged and expressed in folds with respect to GH controls.

### Single fiber gel electrophoresis

The techniques used were introduced previously (Pae et al., 2005). To isolate single fibers, muscle samples were placed in a relaxing solution overnight. The samples were dissected under a microscope (Stereomaster®, Fisher) with micro-tweezers, and then, fibers were mechanically isolated from the non-tendinous end of the sample, and placed directly into 12.5 ul of SDS sample buffer (100 mM Tris Base, 100 mM Tris (pH 6.8), 5% glycerol, 4% SDS, 0.05% Bromophenol Blue, 5% B mercaptoethanol). The single fibers were denatured in the sample buffer and boiled at 100°C for 6 min. MyHC protein isoforms were separated from each fiber dropped in each lane using the SDS-PAGE protocol using a vertical slab gel unit (CBS Scientific, Solana Beach, CA). The separating gel (30% glycerol, 8% total acrylamide (2% Bis), 0.2 M Tris Base (pH 8.8), 0.1 M glycine, 0.4% SDS, 0.1% APS, 0.05% TEMED) was injected between the two plates. The preparation was subjected to 245 volts for 1 h, followed by 375 volts for 24 h. The gel was stained with Coomassie blue G250 for 1–2 h under observation, destained in 10% acetic acid and 25% methanol, and mounted on a drying frame for 24 h. Each stained gel was scanned and captured using Epson Perfection Scanner and Adobe Photoshop software without digital modification.

### Preparation of C2C12 myocytes

C2C12 cells were proliferated for 7~10 days to have myoblasts converted to myotubes which contain more than 2 nuclei in a fiber) in the medium with 10% FBS in DMEM media. When C2C12 was reached 70% confluency, the differentiation medium was changed to DMEM with 2% horse serum. Media was replaced every 2~3 days until the myotubes were harvested. The myotubes were infected with empty vectors or Pdk-1 plasmids (Plasmid 20564; pWZL Neo Myr Flag PDK1, Addgene), and then were exposed to hypoxic conditions. The 60 mm dishes were placed in a hypoxia chamber (Billups-Rothenberg Inc.), and internal gases alternated between room air and a hypoxic gas mixture (1% O2, 5% CO2, and 94% N2) every 10 min, eight cycles to mimic intermittent hypoxia conditioning in tissue levels. For treatment, the cells were maintained in the hypoxic gas mixture for 6 h at the final stage of differentiation.

### Measurements on mRNA concentration for Hif-1α and Pdk-1

Total RNA was isolated using Trizol (Invitrogen) in accordance with the protocol. Extracted RNA was quantified using spectrophotometry at 260 nm absorbance. RNA was converted to cDNA using iScript (Bio-Rad, #170-8890) and each constructed cDNA was diluted 5-fold. Reverse transcriptase was added to PCR mix with SYBR green master mix (Bio-Rad, #170-8880), and gene specific primers. Amplification was performed using LightCycler 480 System (Roche applied science) and the condition was the following: cycle 1 at 95°C for 10 s, cycle 2 at 55°C for 45 s and cycle 3 at 72°C for 10 s. Each cycle iterated 60 times. Published sequences of the primers were used (See below) and cycling conditions and buffer concentrations were optimized for each primer pair, such that amplification of the desired locus is specific with no secondary products. The following primers were used:

    Hif-1α    F: AGTTGCCACTTCCCCACAACGT
                R: AGCACCTTCCACGTTGCTGACT
    Pdk-1    F: ATGAGAATGCGAGACGGCTTTGTG
                R: TAAACGCCTTTGTCTGCATGGTGCT
  MyHC I    F: AGAGGAAGACAGGAAGAACCTAC
                R: CTAAGGATGCCTGTGAAGCC
MyHC IIx    F: AAGACCGCAAGAACGTTCTC
                R: GTCACTCCATTTTGTACTTACGA
MyHC IIa    F: TCCTCAGGCTTCAAGATTTG
                R: GTCCCCATGTGATTCTATTTAA
MyHC IIb    F: GAGGACCGCAAGAACGTG
                R: GGTGACAGAAGAAATCACACA
  β-Actin    F: TCTGAACCCTAAGGCCAACCGTGA
                R: ACCAGAGGCATACAGGGACAACACA

All measurements were normalized by β-Actin. The values were expressed in mean fold changes of standardized mean C_T_ (threshold cycle) values. We used the comparative 2^−ΔΔCt^ method as previously described (Pae et al., 2011). We carried out real-time RT-PCR assays on two sets of tissue harvested from animals.

### Measuring muscle physiological properties using an *in situ* system

Eleven animals were used for muscle fatigue tests. Four control animals and animals treated in sustained hypoxic condition of 15- (*n* = 3) and 30 h-treated (*n* = 4) subgroups were maintained under deep anesthesia by intraperitoneal injection of pentobarbital sodium (80 mg/kg). After the digastric and mylohyoid muscles were detached and severed from the midline, the GH muscle was exposed and isolated, together with the mandible symphysis and the hyoid bone as reported previously (Pae et al., 2005). Briefly, after all infrahyoid and stylohyoid muscles were severed, the lateral and medial branches of the hypoglossal nerve were transected at the distal of the last ramified branches, leaving a collateral of the medial branch intact in the proximal side of the amputation site. This collateral branch solely innervates the GH muscle in rats. The mandible symphysis was secured to the fixture of a custom-designed *in situ* system by using orthodontic wire ligatures, and the mid-sagittal part of the hyoid bone was tied to a force transducer (model FT03, Astro-Med, West Warwick, RI) by using 3-0 silk suture. A skin pouch, filled with mineral oil, was made to provide baths for isolation of the nerve trunks; the trunks were stimulated with a pair of wire electrodes. For *in situ* data collection, the GH muscles were stimulated by using a Grass S48 stimulator (Astro-Med Inc., West Warwick, RI), and forces were assessed using a force transducer and 15LT Bipolar Amplifier System (Astro- Med), together with a desktop computer. After the muscle length was adjusted (L_o_) in order to obtain a consistent tension-output using the baseline voltage (6–9 V), fatigue was induced by stimulation of the hypoglossal nerve at 30 Hz with 300-ms trains every second for 120 s. After the first force output (tension) at L_o_ was determined, peak-force and force-output declines were measured and normalized by expressing the force generated at 30, 60, 90, and 120 s as a percentage of the initial tension measurement at the first pulse. Data acquisition and analyses were performed by a Polyview system (Astro-Med) and LabView (National Instruments, Austin, TX).

### Statistical methods

We used Student *t*-tests for inference test on Western blot results. Number changes in single fiber counts were expressed in percentages and Chi-square tests calculated significance on the differences in fiber compositions between control and two experimental conditions. Triplicate results from quantitative RT-PCR assays were averaged and standard errors (SE) were calculated. We evaluated group differences in fatigability at each data point by using one-way ANOVA (SPSS version 21.0) followed by a *Post-hoc* multiple comparison test with LSD (Least Significant Difference) correction. Means ± standard deviations (SD) were reported for the fatigability tests.

## Results

Animal weights were 339 ± 57 g (mean ± SD). Wet weights of the GH muscle were 0.18 ± 0.04 g (mean ± SD). No significant differences in animal weights or in wet weights of the GH muscle or other muscles emerged between groups. Results of *in vitro* studies were obtained from C2C12 myotubes after 14 days of differentiation.

### Hif-1α and Pdk-1 express differently to different types of hypoxic challenge in different muscles

**Figure 1:**
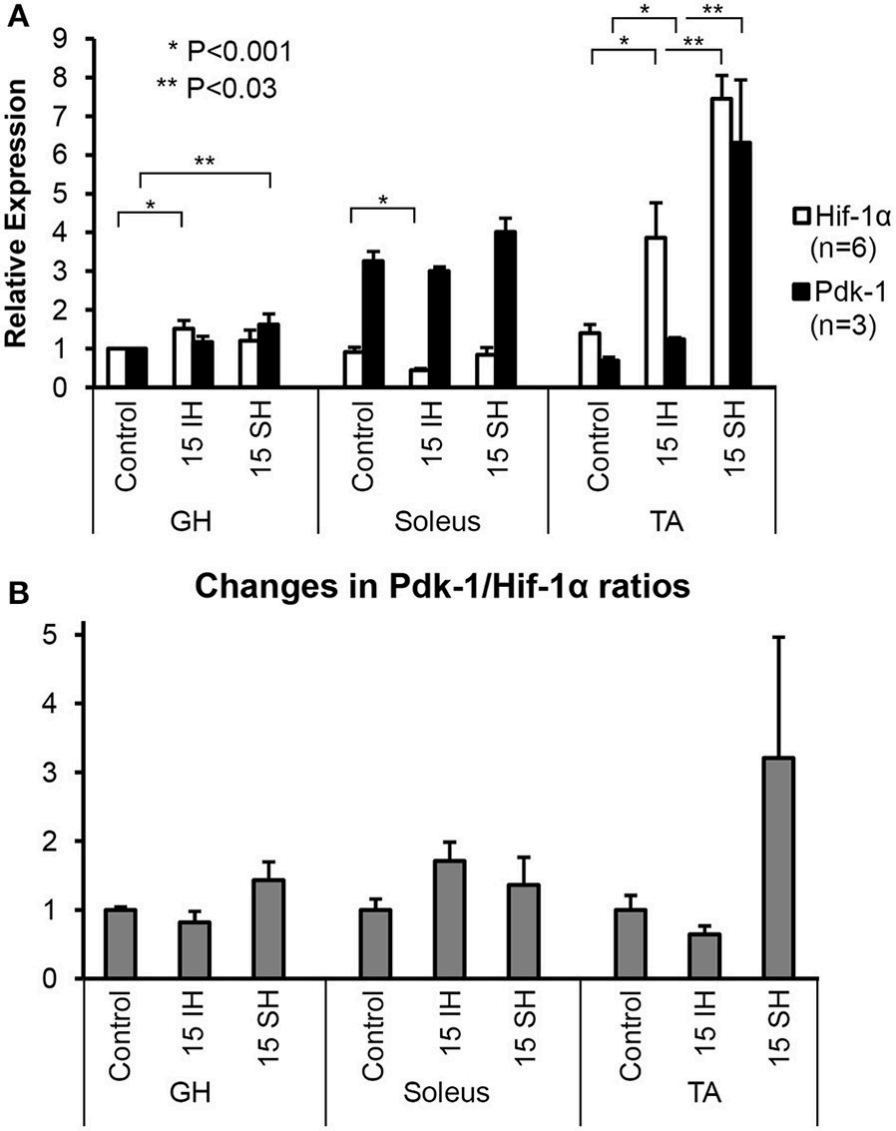
**Fold-expressions of Western blot assay results on GH, soleus, and TA muscles. (A)** Each bar represents the density level of Hif-1 and Pdk-1 relative to the density of control GH muscle. Triplicate measurements were obtained from 2 different animals. Therefore, each measurement indicates an average value of 6 different gels and a corresponding standard error. Comparisons of Hif-1α (detected at 120 kD) and Pdk-1 (detected at 48 kD) expression in ambient air (control), 15 h intermittent hypoxic (IH), and 15 h sustained hypoxic (SH) conditions for GH, soleus, and tibialis anterior. Density levels of Hif-1α and Pdk-1 proteins were first standardized to β-Actin, and then calculated relative to the control GH muscle. Standard errors for each measurement are displayed in error bars. **(B)** Ratios between Pdk-1 and Hif-1α protein concentrations (Pdk-1/Hif-1α) are presented for GH, soleus and TA muscles. The ratios Pdk-1/Hif-1α in hypoxic conditions are standardized to the ratios taken from each control muscle; thus, each ratio measured in control state is used as a denominator. Note that the Pdk-1/Hif-1α ratio was decreased after IH condition and elevated by sustained hypoxia compared to control state in GH muscle.

Hif-1α and Pdk-1 protein levels in the GH, soleus, and TA muscles were analyzed by Western blots normalized with respect to the level of β-Actin as a loading control. Specific signals were detected at 120-kD for Hif-1α antibody and at 48-kD for Pdk-1 antibody (Figure 1A). Density of bands was measured and described using densitometry. For the GH muscles, Hif-1α expression increased by approximately 1.5-fold (1.5 ± 0.87) after 15 h of intermittent hypoxic treatment and 1.2-fold (1.2 ± 0.11) after sustained hypoxic treatment, compared to the control. Relative to the control, Pdk-1 expression was elevated 1.6-fold (1.6 ± 0.15) after 15 h sustained hypoxic exposure. In the predominantly-oxidative soleus muscles, Hif-1α expression was less than that in the GH and the TA muscle under hypoxic conditions. In soleus muscles, 15 h intermittent hypoxic exposure resulted in a decline in Hif-1α expression to an half. The control level of Pdk-1 was highest in the soleus muscle, compared to the control GH and TA muscles. Up to a 3.8-fold (3.8 ± 0.37) increase of Hif-1α level was observed after intermittent hypoxic exposure in the predominantly glycolytic muscles TA, and a 7.4-fold (7.4 ± 1.70) increase was observed in the sustained hypoxia of TA muscles. Pdk-1 expression in the TA measured also highest (6.3-fold) in 15 h sustained hypoxia. Changes in the soleus were led by an increase in Pdk-1 protein, yet changes in the TA were dominated by an increase in Hif-1α irrespective of hypoxia types. We compared Pdk-1/Hif-1α ratios between control and hypoxic conditions. In doing so, we used the ratios in normal conditions as a denominator; thus, each control becomes 1 for each muscle (Figure 1B). When exposed to intermittent hypoxia, soleus muscles increased the Pdk-1/Hif-1α ratio as much as twice; however, sustained hypoxic exposure elevated the Pdk-1/Hif-1α ratio in all three muscles.

### MyHC composition altered in the geniohyoid muscle after sustained hypoxia

**Figure 2:**
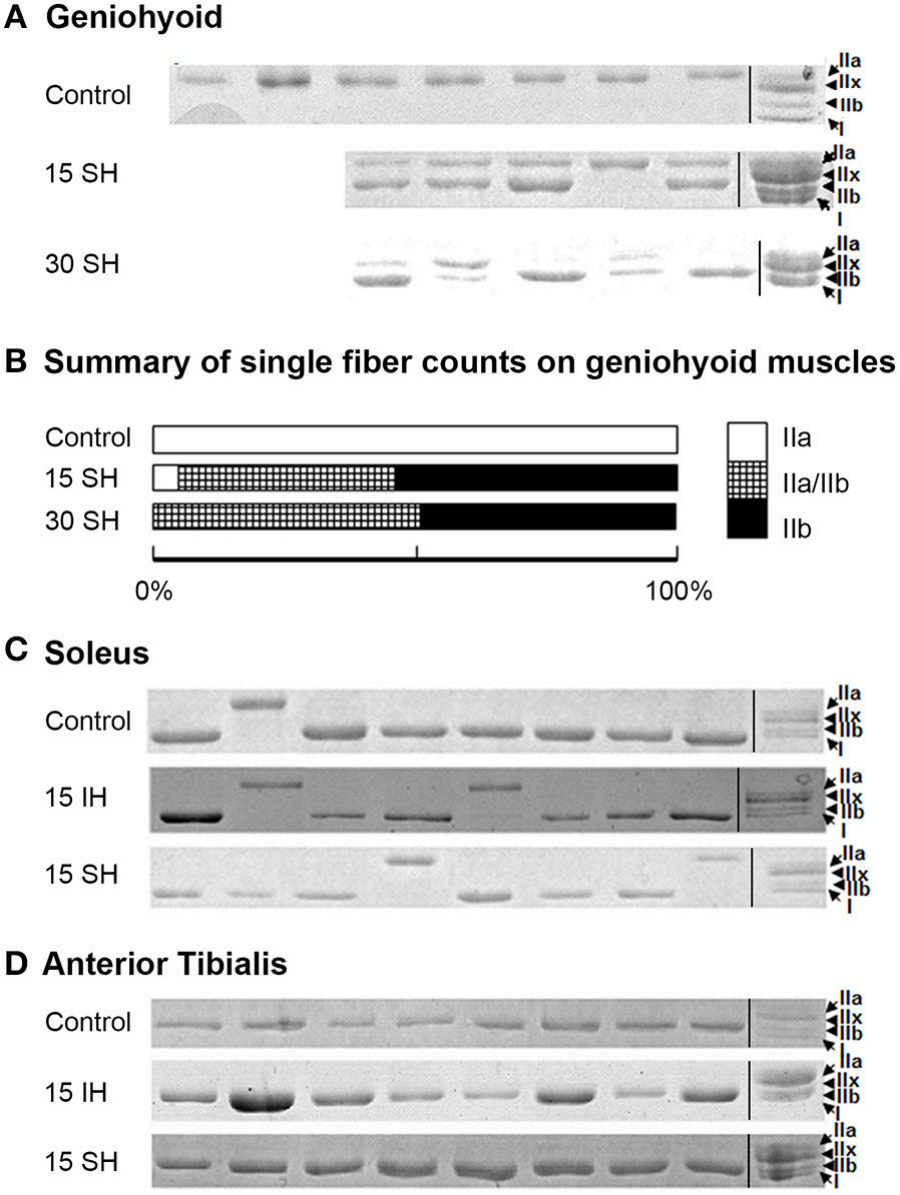
**Electrophoresis on harvested single fibers of the geniohyoid, soleus, and anterior tibialis muscles. (A)** SDS-PAGE gels for single fibers obtained from the GH muscle treated in 15 and 30 h sustained hypoxia. Reference bands for myosin heavy chain isoforms indicate, from the top MyHC IIa, IIx, IIb and I, in the last lanes as a ladder. Note that two bands in each lane for MyHC IIa and IIb proteins are clearly visible after sustained hypoxic conditioning. **(B)** The change in MyHC composition compared to the previously reported control (MyHC IIa) was statistically significant (*P* < 0.05, *n* = 80). **(C)** Control soleus muscle consists of mostly MyHC I-containing fibers with a few MyHC IIa-containing fibers. MyHC composition of the soleus muscle remains unchanged under both intermittent and sustained hypoxic conditions. **(D)** All single fibers harvested from TA muscles consisted of MyHC IIb containing fibers. MyHC composition of the TA muscle remains unchanged in both hypoxic conditions.

Previously, we have reported that the GH muscle in *Sprague Dawley* rats consists of fibers containing MyHC IIa in normoxia (Pae et al., 2005). SDS-PAGE gels for single fibers obtained from the GH muscle are shown in Figure 2. Reference bands for the MyHC isoforms indicate, from the top, MyHC IIa, IIx, IIb and I, in the last lanes. 15 and 30 h sustained hypoxia-treated GH muscle fibers showed co-expression of both IIa and IIb (Figure 2A). Thirty-eight percent of the 15 h treated muscle showed co-expression of IIa and IIb, 52% contained IIb only, and only 8% contained IIa MyHC only (Figure 2B). However, after 30 h sustained hypoxic treatment, 50% of fibers harvested from the GH muscle contained IIa/IIb combination and the rest was balanced by IIb MyHC fibers. When all 80 clearly stained single fibers were counted for statistical analysis, MyHC composition changes of the GH muscles were associated with experimental conditions (i.e., sustained hypoxia) significantly (χ^2^ = 10.34 at Degree of freedom of 4 for control, 15 vs. 30 h sustained hypoxia) at *P* < 0.05.

The two leg muscles, soleus and TA did not show significant changes in composition after hypoxic challenges (Figures 2C,D). Soleus muscles consisted of mainly MyHC I, and occasionally, IIa fibers (Figure 2C), and TA muscles were mostly IIb fibers in control and 15 h treated groups for both intermittent and sustained hypoxia (Figure 2D).

### Exogenous over-expression of Pdk-1 protein using plasmids significantly alters MyHC expressions in C2C12 myotubes

**Figure 3:**
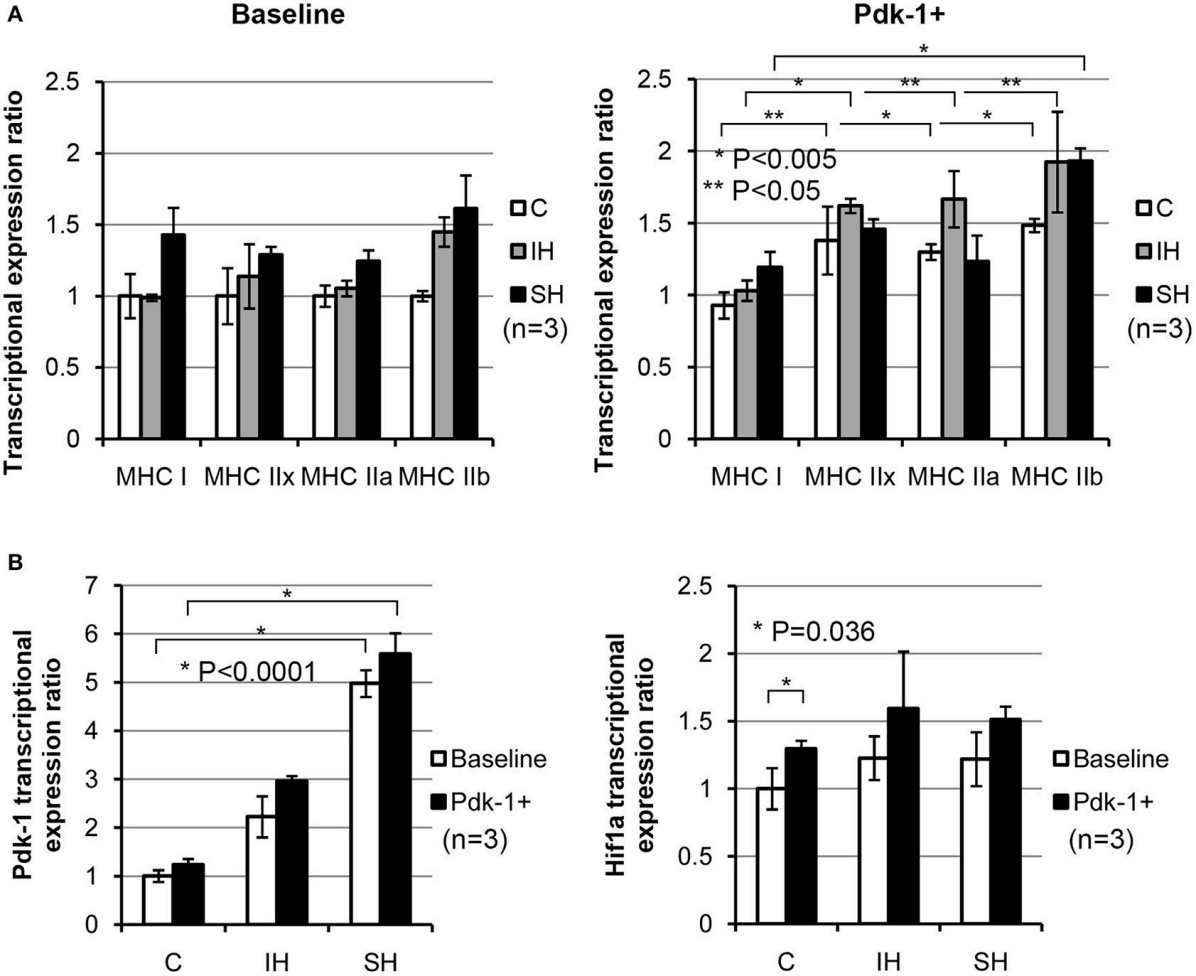
**(A)** Note shifted MyHC mRNA expressions toward glycolytic side in IH and SH hypoxia after Pdk-1 protein was augmented. mRNA expression for each MyHC after Pdk-1 over-expression was compared to the baseline state in control muscle. For MyHC IIx and IIb, each comparison between Baseline vs. PDK+ were statistically significant. MyHC IIb increased most, but IIx and IIa also increased. For MyHC IIx, 1.0 ± 0.19 vs. 1.37 ± 0.23 under control, 1.13 ± 0.22 vs. 1.62 ± 0.04 under IH, and 1.28 ± 0.05 vs. 1.45 ± 0.06 under SH were measured. For MyHC IIb, 1.0 ± 0.03 vs. 1.48 ± 0.04 under control, 1.44 ± 0.10 vs. 1.92 ± 0.34 under IH, and 1.61 ± 0.23 vs. 1.93 ± 0.08 under SH were measured. All comparisons (means ± SE, *n* = 3) differ significantly at least at *P* < 0.05. **(B)** Changes in Pdk-1 and Hif-1α mRNA concentrations in C2C12 myotubes in hypoxia. Effects of hypoxic conditions on the expression changes are significantly greater than those changes resulted from Pdk-1 augmentation in Pdk-1 measurements. After Pdk-1 was overexpressed in myotubes, Hif-1α mRNA expressions did not significantly change in either IH or SH condition.

Using plasmid-mediated methods, Pdk-1 protein was over-expressed in C2C12 myotubes. Compared to the myotubes in baseline (Figure 3A), expressions for MyHC IIx, IIa, and IIb in the control condition (C, white bars) were increased significantly after addition of Pdk-1 (Pdk-1+). After IH conditioning (Gray bars), MyHC for IIx, IIa, and IIb isoforms increased. However, MyHC IIb only increased in sustained hypoxia (Black bars) when Pdk-1 was over expressed.

### Differential expressions of Pdk-1 and Hif-1α emerge in accordance with types of hypoxia

When the results of Pdk-1 over-expression versus hypoxia effects were evaluated, the differential effects from the mode of hypoxia were much stronger than Pdk-1 over-expression (Figure 3B). Particularly, the mRNA expressions of Pdk-1 after sustained hypoxia increased markedly in Baseline and Pdk-1+ myotubes. However, when Hif-1α expressions were measured, Hif-1α expressions elevated only nominal degrees in both myotubes in baseline and treated with Pdk-1 plasmids after both hypoxic challenges.

### Fatigability of the geniohyoid muscle improves after sustained hypoxia

**Figure 4:**
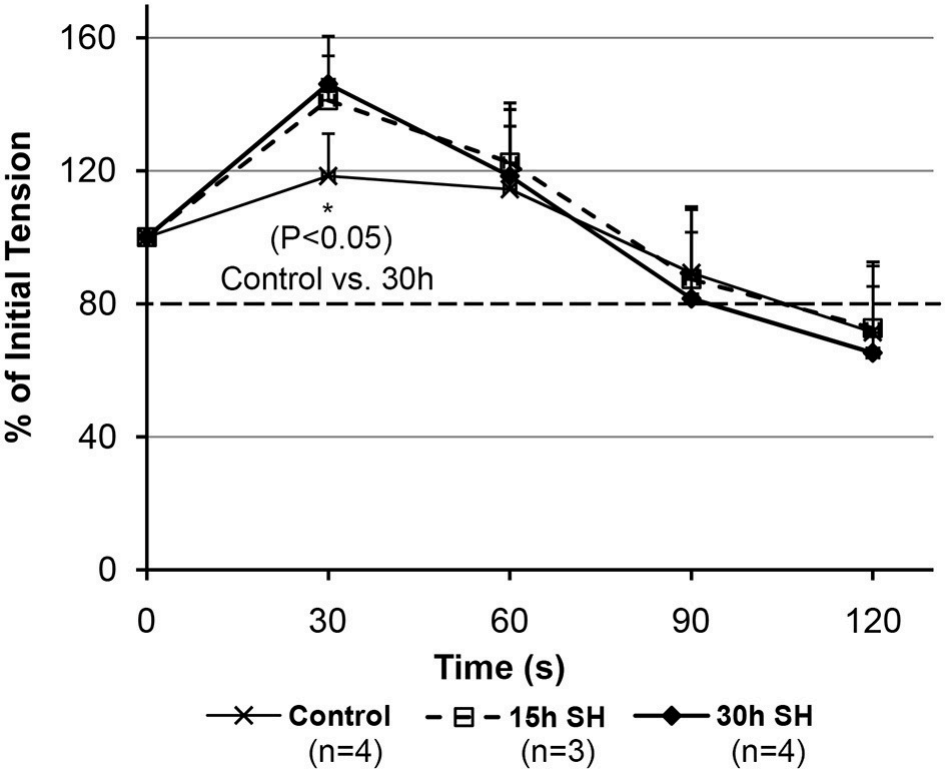
**Fatigability measurements on the geniohyoid muscle after sustained hypoxic challenges**. When muscle tension force produced by the GH muscles were compared between Control vs. 15 vs. 30 h at each time-point, 30 s-point only showed a significant difference between Control and 30 h (Control 118.37 ± 12.81 vs. 30 h 146.09 ± 8.54, *P* = 0.035, where mean ± Standard Deviation).

We observed initial potentiations at 30 s time-point (Figure 4). The rats treated in sustained hypoxia for 30 h only, however, showed a significantly increased tension (*P* = 0.035) at 30 s-point compared to that of control animals (Control, 118.37 ± 12.81 vs. 30 h, 146.09 ± 8.54). 15 h treated animals showed 118.77, 136.53, and 167.12% at 30 s-point compared to the initial tension measured in each animal. The elevation of initial potentiation in the animals treated under 15 h sustained hypoxia was not significant (*P* = 0.250) at 30 s (140.80 ± 24.45) compared to those of the controls.

## Discussion

Short-term intermittent hypoxia (IH) occurs commonly in patients with respiratory disorders. We have previously reported IH alters fiber-type composition and physical characteristics of a hyoid muscle (Pae et al., 2005). It is well known that prolonged IH induces atrophy and increased fatigability in skeletal muscle due to ROS (Powers et al., 2011; Kozakowska et al., 2015). However, molecular responses to short-term sustained hypoxia appeals little interests, thus is not well understood. We hypothesized that the geniohyoid and limb muscles adapt differently to hypoxic challenges because hypoxia stimulates ventilation which triggers an exercise-stimulus concomitantly in the hyoid muscle.

Chronic exposure to hypoxia results in a decreased oxidative capacity of the skeletal muscle. This alteration accompanies up-regulation of a hypoxia marker such as Pdk-1 that is a molecule blocking pyruvate from being transferred into the TCA cycle (Hoppeler et al., 2003; De Palma et al., 2007). Physical training in a chronic hypoxic condition gradually improves oxygen transfer capacity *via* increased capillary numbers and amount of myoglobin in the muscle. In fact, acclimatization to hypobaric hypoxia brings Hif-1 and Pdk-1 levels back to pre-hypoxic levels in 7–9 days after removal of the hypoxic condition (Viganò et al., 2008). Therefore, we think that there may be an acute adaptation mechanism for homeostasis in an initial stage of the process also.

Geniohyoid (GH) muscles are frequently used for hypoxia studies due to a constant anatomical position and measurable length in rat model, and are easily accessible for physiological measurements (Holman et al., 2012). The GH muscle originates from the inferior genial tubercle of the mandible and inserts into the hyoid bone. The GH muscle stabilizes the hyoid bone position to open the airway as holding the hyoid bone in a static position (Wiegand et al., 1990; Pearson et al., 2011). Skeletal muscles are primarily post-mitotic tissue with little turnover once matured except for regeneration *via* satellite cells after injury (Brack and Rando, 2012); thus, most structural changes of this tissue are accomplished *via* altering the genes expressed in already existing fibers (Gundersen, 2011). We previously observed an intermittent hypoxic challenge convert the geniohyoid (GH) muscle composition toward fast-twitch fibers in 5 h (Pae et al., 2005). We assumed a different glycolytic change in the same muscle after sustained hypoxic exposure in proportion to the length of hypoxia. Such adaptive phenotypic changes in a single fiber have well demonstrated previously (Caiozzo et al., 2003).

Our previous study in rats demonstrated that intermittent hypoxic challenge, which often accompanies a rather common disease, obstructive sleep apnea, adversely affects the endurance of the GH muscle. A long-term endurance training of the GH muscle could help improve symptoms of OSA which of pathophysiology is often attributed to fatigable upper airway dilators (Petrof et al., 1994). Our study demonstrated polymorphism appeared in the GH muscle as early as 5 h of intermittent hypoxia treatment, and by 10 h, all fibers converted from fatigue- resistant fast types (MyHC IIa) to fatigue-vulnerable fast forms (MyHC IIb) (Pae et al., 2005). The fast disappearance of fibers containing MyHC IIa, is often attributed to oxidative stress which could result in mass reduction and a decrease of mitochondrial volume density when exposed for months (Muller et al., 2006). In this study, we did not observe a difference in mass of the GH muscle between the groups, but GH muscle fiber composition change from IIa to IIa/b were significant in sustained hypoxia in conjunction with expression changes of Pdk-1 proteins.

### Fatigue resistant MyHC IIa protein-containing fibers are preserved in sustained hypoxia

In contrast to the IH challenged muscles which lost all MyHC IIa fibers in 15 h exposure, the current sustained hypoxia-challenged GH muscles clearly showed an improvement in fatigability and a preservation of the original MyHC IIa containing fibers and a significant change in fiber composition from MyHC IIa-dominant to a MyHC IIa/IIb-combination. These findings may indicate an adaptive response of a “mixed-type” skeletal muscle to sustained hypoxia. Adaptation process of skeletal muscles to hypoxic environment is slow and often results in insignificant changes. Acute hypoxia, such as exercising in hypoxia, readily increases tissue protein levels of Hif-1 and Pdk-1, but an adaptation follows immediately (Le Moine et al., 2011). Hif-1 signaling in a hypoxic muscle induced by ROS generated from mitochondria has a tendency to sustain the level of ROS for metabolic homeostasis; thus, Hif-1 activates Pdk-1 to modulate pyruvate dehydrogenase activity (Kim et al., 2006; Viganò et al., 2008; Cerretelli and Gelfi, 2011).

Our C2C12 data showed that Pdk-1 levels in the myotubes under sustained hypoxia up-regulated significantly (Figure 3B) as demonstrated in the GH muscle (Figure 1). Upregulated Pdk-1 possibly down-regulates ROS as well as decreases oxygen consumption for homeostasis. In fact, an amplification of Pdk-1 with respect to Hif-1 levels favors; first, increased availability of oxygen and second, decreased cell death (Papandreou et al., 2006). This explanation supports our observations how MyHC IIa fibers survived in sustained hypoxia (in this study), but did not in intermittent hypoxia (in previous studies).

### Differential expressions of Hif-1α and Pdk-1 were evident in different hypoxic conditions in the same muscle

The current results showed that the two distal limb muscles, the soleus (a “slow” muscle mainly with oxidative fibers) and the anterior tibialis (a “fast” muscle mainly with glycolytic fibers), did not result in a change in MyHC isoforms under either acute intermittent or sustained hypoxic exposure (Figure 2). A previous report on fiber composition changes of the soleus muscle under intermittent hypoxia was similar (McGuire et al., 2003). As reported in this study and other studies (Pisani and Dechesne, 2005), soleus muscles contain a low amount of Hif-1α while TA muscles contain a high Hif-1α. However, the amount of Pdk-1 in the soleus muscle in control state appears higher than the other two (Figure 1). Contrarily, we noted that the amount of Hif-1α in the TA muscle increased markedly which may able maintaining characteristics of those fibers consisting of the TA muscle (Figure 2). Our observation prompts following assertions: first, physiological responses to sustained hypoxia may differ in the limb from hyoid muscles. Second, fiber-type conversion observed in the GH muscle in hypoxic conditions may be induced by compensatory breathing efforts for more oxygen intake, as well as by interactions with other oxygen-sensing molecule induced by sustained hypoxia such as Pdk-1 which may take an advantage over Hif-1α in their reaction cascades as shown in myotubes (Figure 3B).

Hif-1 may provide an explanation at least partially for underlying mechanisms for muscle fiber type changes in hypoxia (Allen et al., 2001; Semenza, 2007). As a main player to restore oxygen homeostasis at cellular and tissue levels, Hif-1α expression is observed to increase in response to systemic hypoxia (Stroka et al., 2001) and high frequency muscle contraction (Lunde et al., 2011). We confirm that the baseline level of Hif-1α in the control groups of the tibialis anterior muscles were higher than those in geniohyoid muscles, and in turn, higher than the soleus muscles; all may suggest that Hif-1α could have potential functions that are not limited to muscle oxygen sensing and homeostasis. Lunde et al. (2011) explained a difference in the Hif-1 signaling system between fast and slow muscles. They suggested that Hif-1α may be strongly suppressed post-transcriptionally in slow muscles *via* a more active degradation of von Hippel-Lindau (Vhl) protein acting in the upstream. It appears that the level of Hif-1α mRNA in normal resting muscles often does not necessarily correlate with the level of Hif-1α proteins (Huey and Bodine, 1998; Reilly et al., 2000; Mounier et al., 2010) during function or in hypoxia.

### Pdk-1 likely counterbalances activity of Hif-1, and that is respiration-type dependent

Pdk-1 expression in Hif-1α null myoblasts in response to hypoxia (0.5% oxygen) is attenuated compared to the upregulation in wild types (Mason et al., 2007). Thus, Hif-1α is an upstream molecule regulating Pdk-1 in hypoxia. During hypoxia, cytoplasmic Hif-1 regulates many target genes involved in glycolysis, angiogenesis, and cell survival. A modulation of glucose metabolism starts pyruvate, which can either be converted into lactate by lactate dehydrogenase A or into acetyl-CoA by the mitochondrial enzyme pyruvate dehydrogenase, which is inhibited by Pdk-1 under hypoxia in a Hif-1-dependent manner (Kim et al., 2006). Therefore, in order for a glycolytic muscle to be maintained, glycolytic-favorable conditions must be preserved as shown in the case of the tibialis anterior muscle, regardless of the level of Hif-1α, Pdk-1 expression, or the type of hypoxic (sustained or intermittent) condition.

In hypoxia, Hif-1α protein level in the geniohyoid muscle was higher than room air exposure, which was also found in other studies (Peng et al., 2006). The level of Pdk-1 expression relative to the level of Hif-1α was higher in geniohyoid muscles treated under sustained hypoxia than with intermittent hypoxia for Hif-1 level increased significantly with intermittent hypoxia (See Figure 1). This observation may provide insights as to why the original type IIa fibers were retained in sustained hypoxic condition, but not in the intermittent hypoxic animals as previously shown. It has been suggested that intermittent hypoxia increases ROS as increases Hif-1 level for intermittent hypoxia resembles ischemia-reperfusion (Chandel et al., 2000; Clanton, 2007). Increased ROS levels in hypoxic condition activate the signaling mechanism led by a pro-inflammatory transcription factor NFκB (Ryan et al., 2007; Osorio-Fuentealba et al., 2009), and may trigger apoptosis in mitochondria (Balaban et al., 2005), and in turn, result in cell death. In fact, repeated periods of partial hypoxia induces histomorphologic damage with inflammation, and results in apoptosis in rat skeletal muscle (Aravindan et al., 2007). Apoptosis resulting from increased Hif-1 concentration higher than Pdk-1 might have resulted in an elimination of the oxidative type IIa fibers in the GH muscle in intermittent hypoxia (Pae et al., 2005). In such sense, Pdk-1 level is crucial for maintaining production of ATP levels and for attenuation of ROS (Kim et al., 2006; Papandreou et al., 2006; Yeluri et al., 2009) produced in mitochondria. For this reason, the slightly increased level (35%) of Pdk-1 in the GH muscles under sustained hypoxia (See Figure 1) may serve as a counterbalancing (or dampening) mechanism toward ROS for the maintenance of the original fatigue-resistant MyHC IIa containing fibers, while favoring the formation of new glycolytic type IIb fibers. In addition, when Pdk-1 was added to the myotubes, Hif-1α levels were attenuated significantly regardless of hypoxia types (See Figures 3A,B under Pdk-1+).

Hif-1α levels in predominantly oxidative muscles are lower than those in predominantly glycolytic muscles (Pisani and Dechesne, 2005); on the other hand, Pdk-1 expression is much higher in oxidative, compared to glycolytic muscles (Peters et al., 2001; LeBlanc et al., 2007). In agreement with previous studies, while basal levels of Hif-1α expression in the soleus muscle were lower than those of GH and tibialis anterior, Pdk-1/Hif-1 ratios maintain greater than 1 in both intermittent and sustained hypoxia in our study (See Figure 1B). Therefore, a low concentration of Hif-1, in conjunction with a high concentration of Pdk-1 may contribute to the minimal change of soleus fiber composition. In addition, literature demonstrates that ROS removal capacity of the type I fibers is known to significantly greater than that of type IIb fibers (Anderson and Neufer, 2006). With the high level of Pdk-1 which did not change significantly under hypoxic conditions and possibly served as an aforementioned dampening mechanism against produced ROS. As shown in Pdk-1 augmented myotubes, a hypoxic condition regardless of the type favors glycolytic fibers (Figure 3A). A magnitude of Pdk-1 amplification responding to Hif-1α in the skeletal muscle appears to influence the composition of muscle fiber types in a mixed type muscle. In other words, Hif-1 does not directly regulate glucose metabolisms but via Pdk-1. A fatigable mixed type muscle could be a fatigue resistant mixed type by means of Pdk-1.

### Caveat

The tension (force-output) measured on the GH muscles treated in sustained hypoxia challenges maintained above our tentative 80% cutoff line indicated by a darker broken line in Figure 4 (88% for 15 h and 82% for 30 h at 90 s). As shown in Figure 4, 30 h animals demonstrated a declining average tension sharply between 60 and 90 s time-points in comparison to the control and the 15 h-treated animals. As a result, comparisons on the tension measurements at 90 s between the groups did not reveal a statistical significance. However, at 90 s, all groups maintained their average tensions above the “80% line” on the graph, which indicates a comparable endurance. The activity-dependent potentiation did result in the greater isometric force (i.e., potentiation) for the submaximal stimulation. However, fatigue also resulted in a less tension after a longer (30 h) hypoxic exposure whereas the shorter 15 h group sustained their tension outputs as much as the control animals did. Therefore, there appear two opposing processes occurring at the same time in the muscle, i.e., one that enhances muscle performance through fiber type modulation and one that decreases muscle performance probably due to depletion of calcium concentration during repetitive stimulation.

## Conclusion

In summary, our results suggest that hyoid muscles respond differently in sustained from intermittent hypoxia, and that alteration in muscle physiological characteristics may be commensurate with, not decoupled from, a switch in fiber composition. Our observations do not offer direct evidence that the ratio Pdk-1/Hif-1α modulates muscle fiber type change in the GH muscle, but suggest an adaptive, so protective mechanism to hypoxic challenge primarily by Pdk-1. Caveats of the current report may include a lack of mechanistic *in vivo*-data demonstrating a dose-effect relationship between Pdk-1 level and MyHC expression changes. Future studies that quantify and manipulate the level of Pdk-1 and Hif-1 in not C2C12 myotubes but primary specific myotubes from oxidative and glycolytic muscles may help understand the mechanism underlying disappearance of IIa fibers after intermittent hypoxic insult, and their appearance in a sustained hypoxic condition. Our results suggest that a mixed-type, GH muscle, may convert into less fatigable MyHC abundant after acute sustained hypoxic conditioning *via* a role of protein Pdk-1. This imposes a potential utility for improving medical conditions in humans such as OSA and chronic obstructive pulmonary disease. We conclude that Pdk-1 protein has an additional role protecting existing fibers in addition to the role driving fibers toward glycolytic expression.

## Author contributions

All authors listed, have made substantial, direct and intellectual contribution to the work, and approved it for publication.

### Conflict of interest statement

The authors declare that the research was conducted in the absence of any commercial or financial relationships that could be construed as a potential conflict of interest. The reviewer CC and handling Editor declared their shared affiliation, and the handling Editor states that the process nevertheless met the standards of a fair and objective review.
